# Supplementary material for: Dynamic Phenotypic Switching and Group Behavior Help Non-Small Cell Lung Cancer Cells Evade Chemotherapy
Source: Biomolecules. 2021 Dec 21;12(1):8. doi: 10.3390/biom12010008 (PMC8773639; doi:10.3390/biom12010008)
Supplement: Supplementary file 1 [file biomolecules-12-00008-s001.zip › Combined Supp Figures Tables and Legends final.pdf]

Supplementary Figure S1

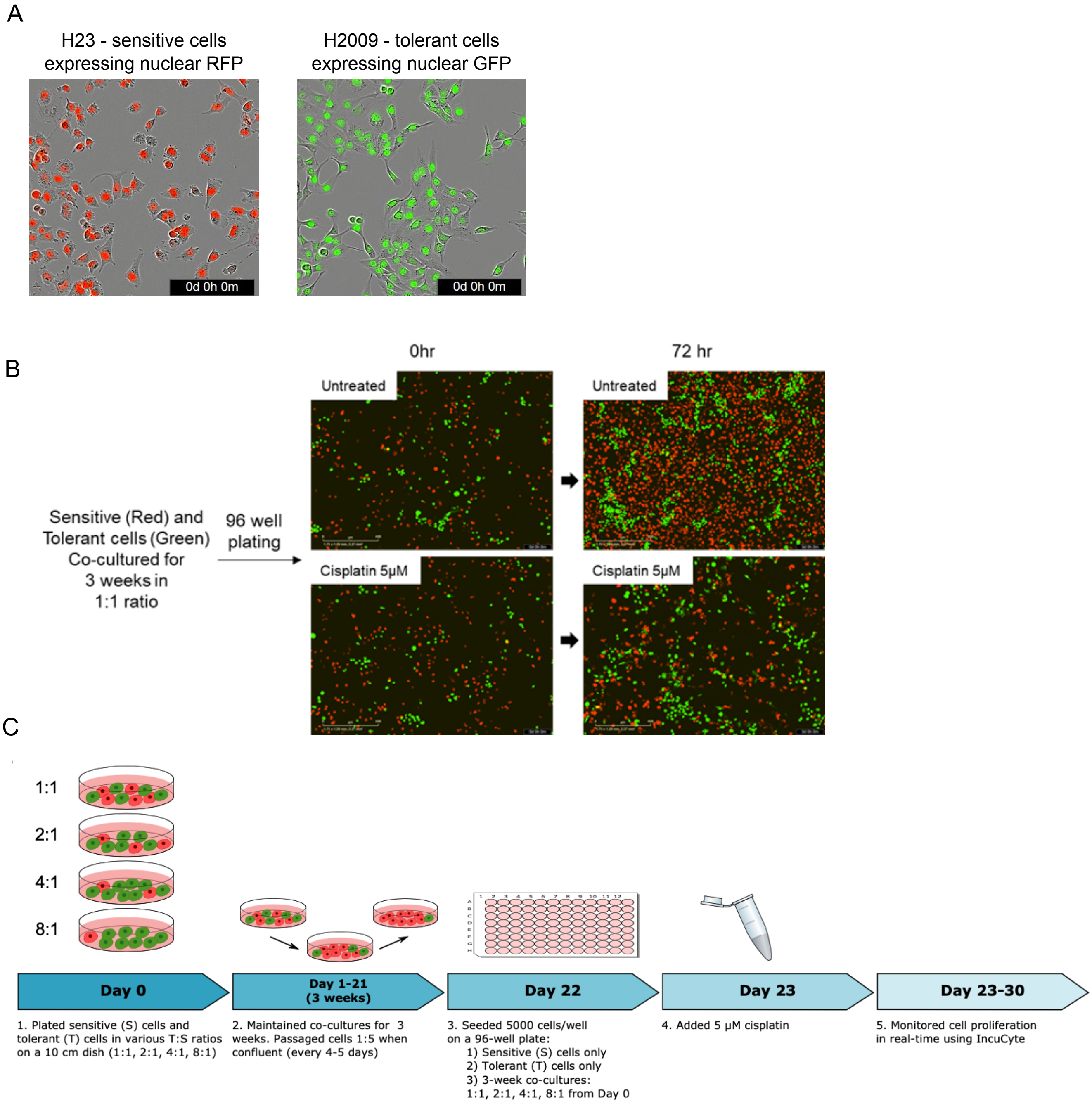

Supplementary Figure S2

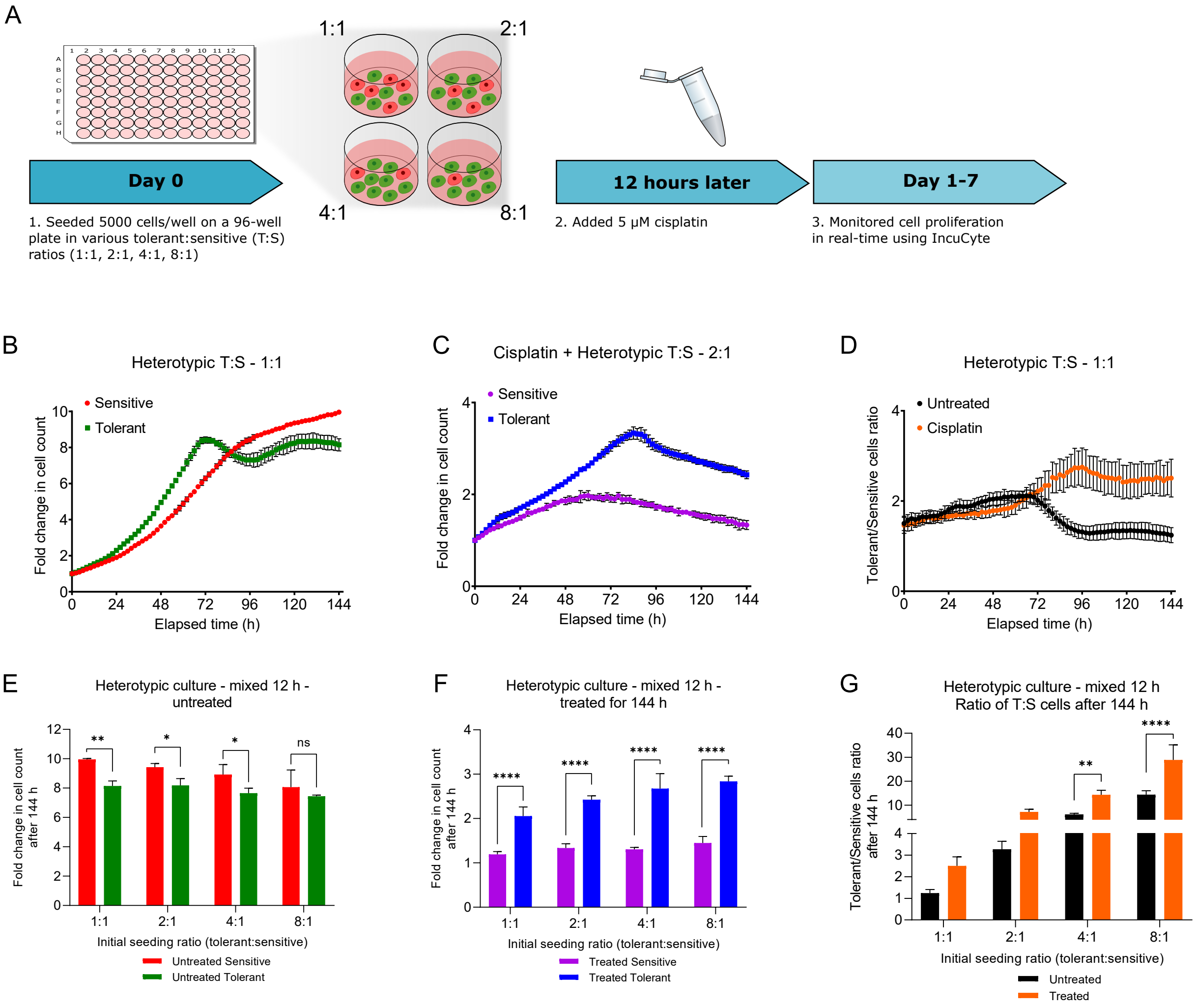

Supplementary Figure S3

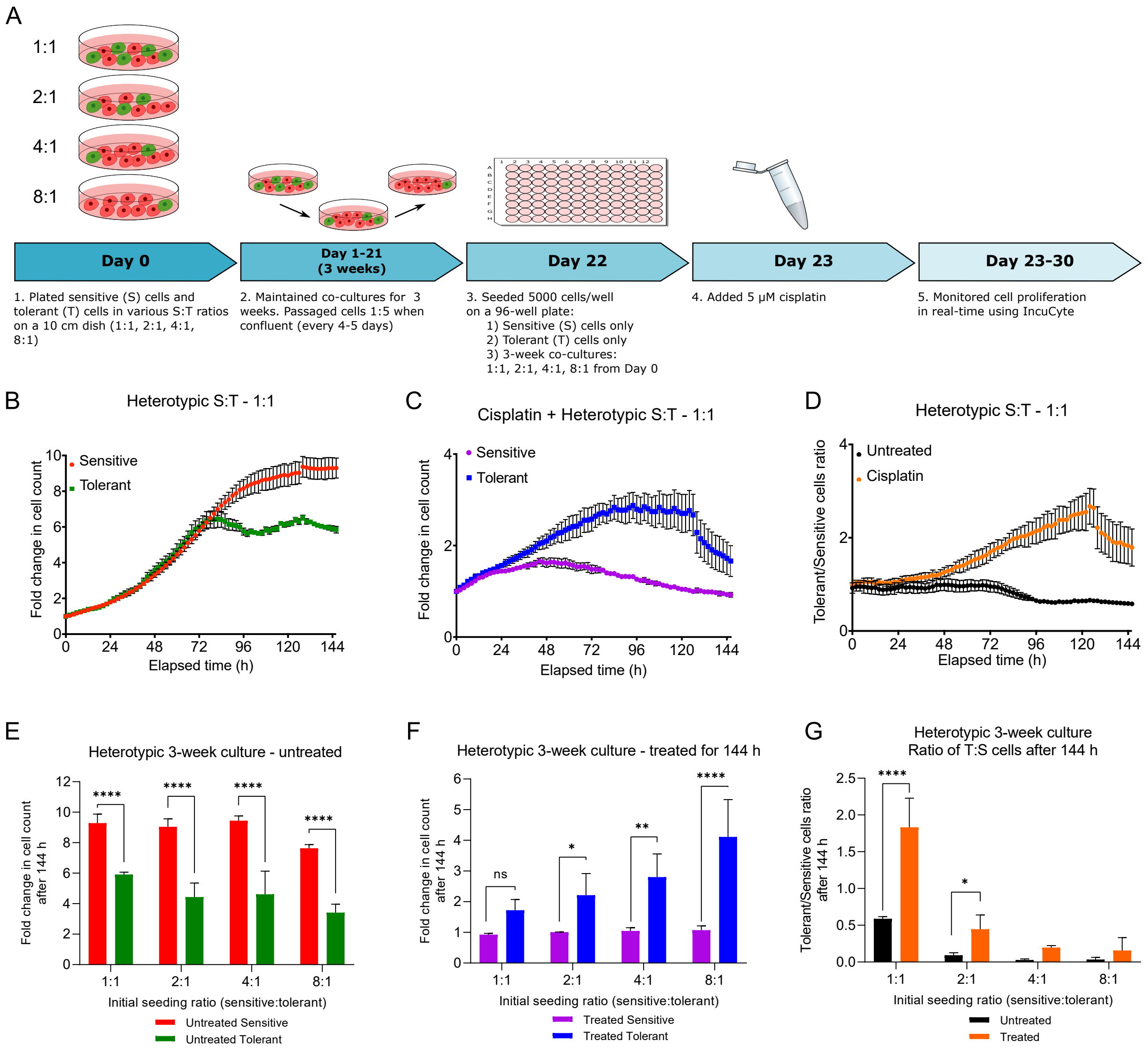

Supplementary Figure S4

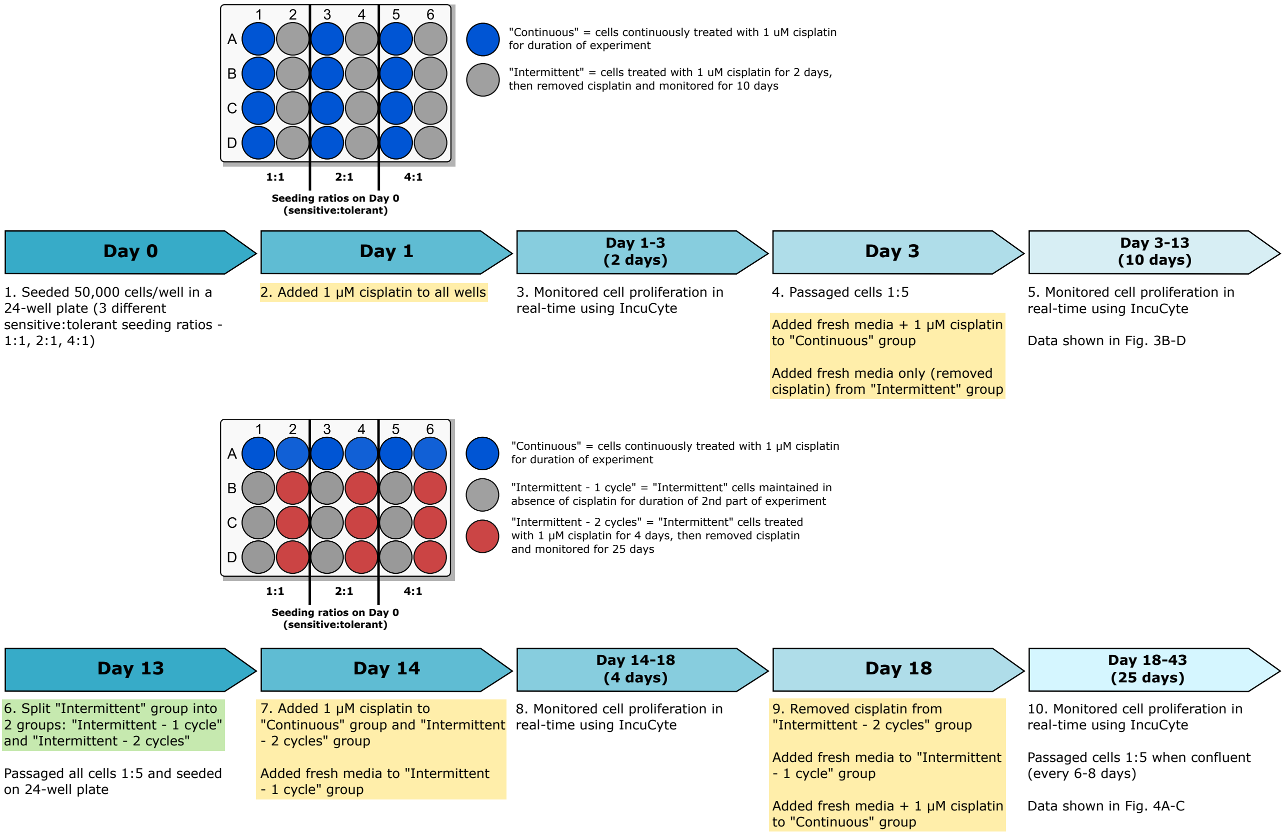

Supplementary Figure S5

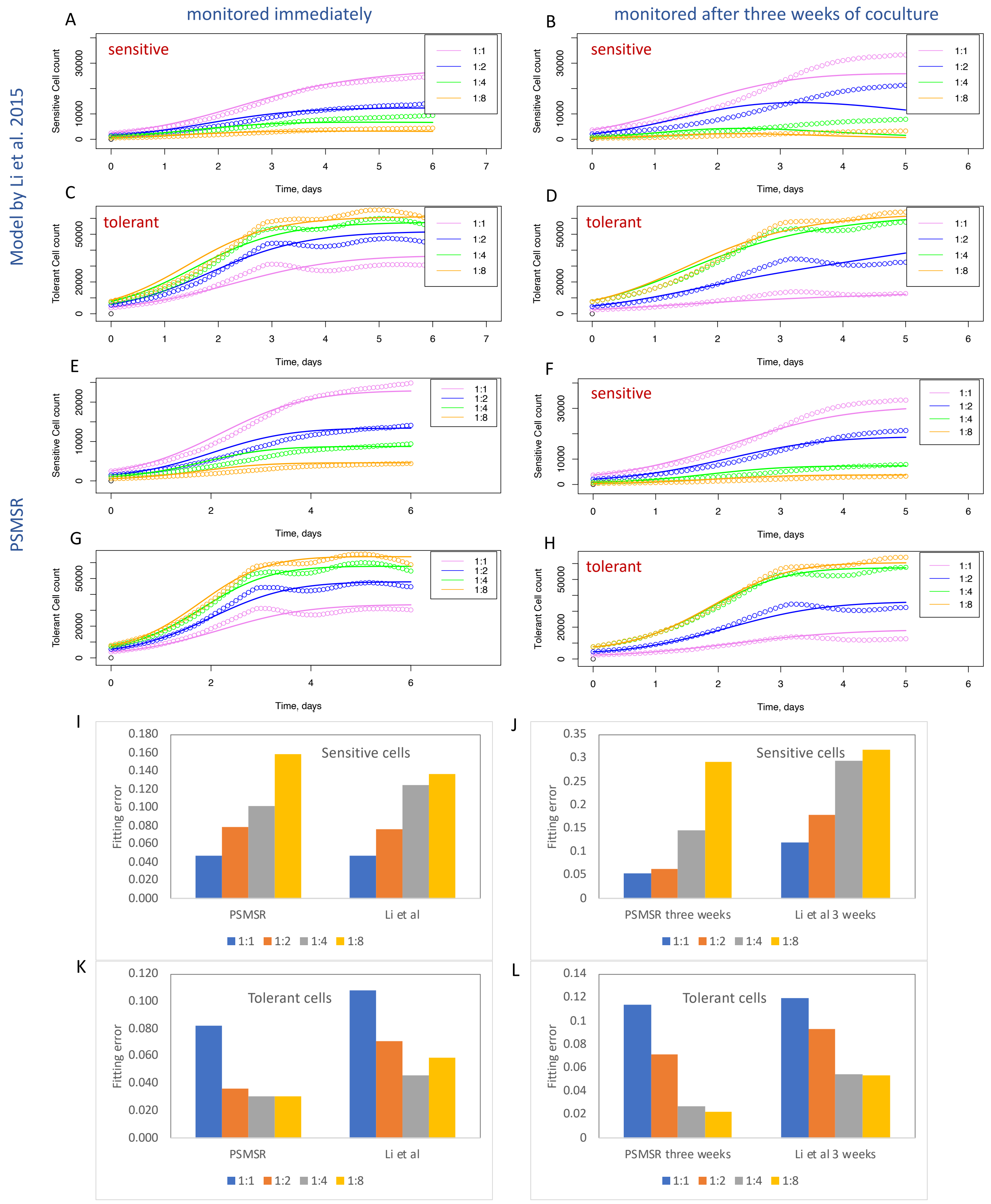

Supplementary Figure S6

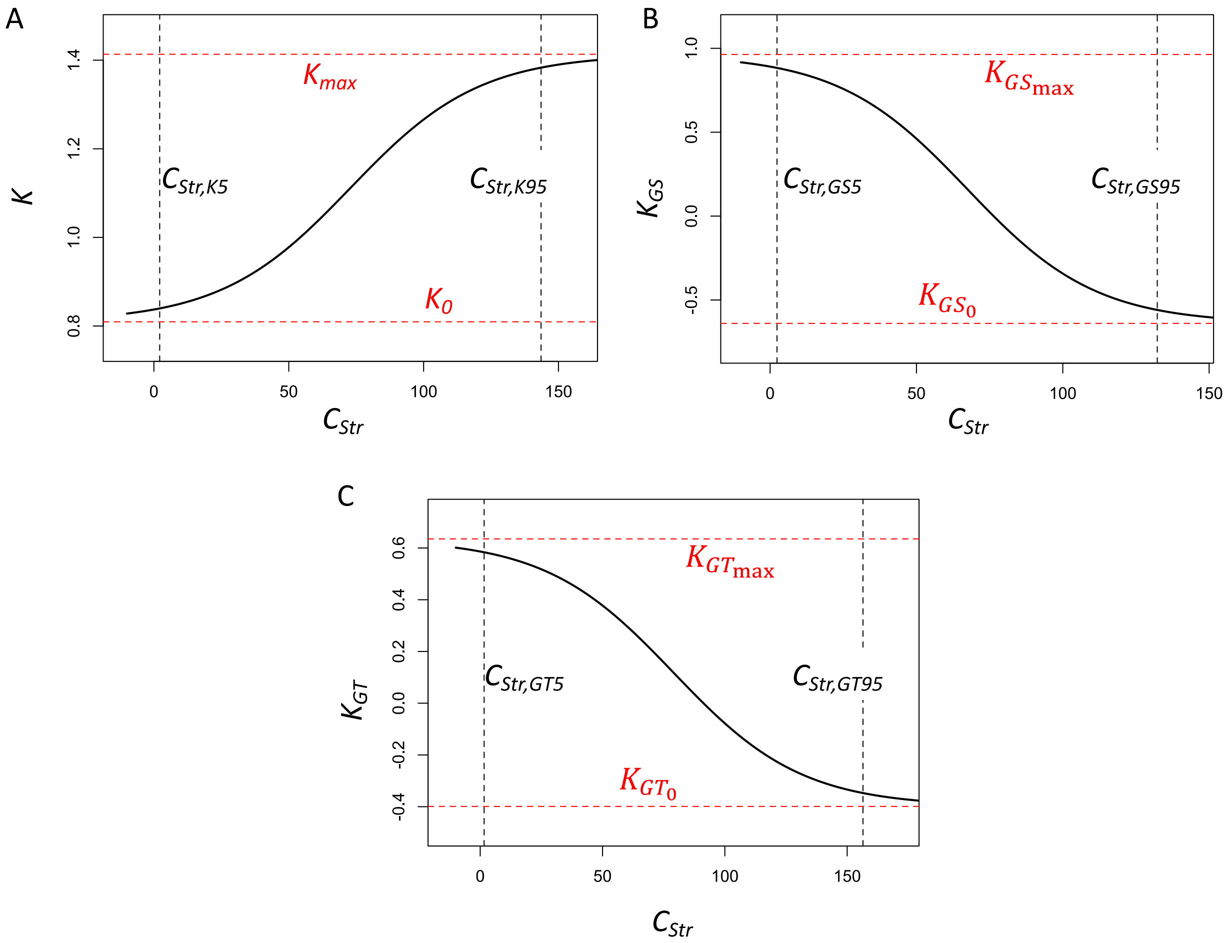

Supplementary Figure S7

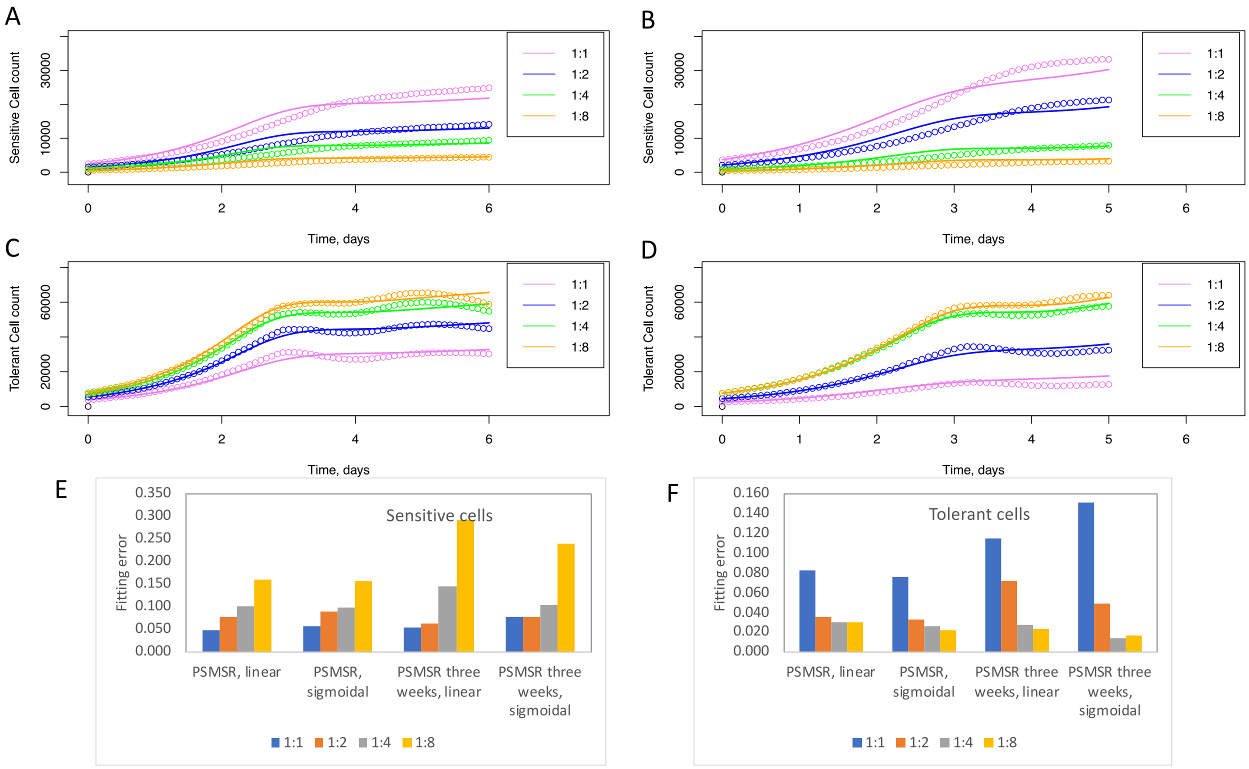

Supplementary Figure S8

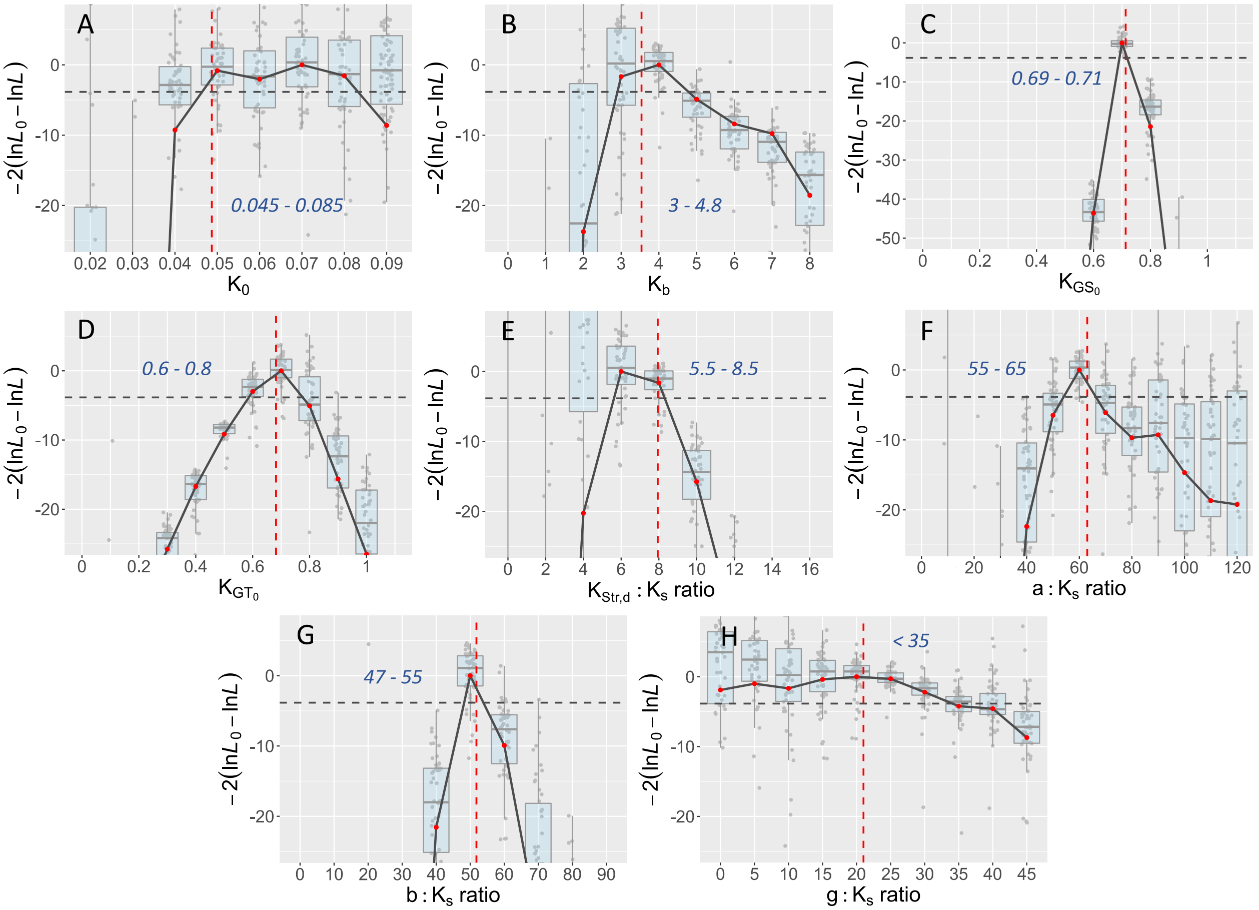

Supplementary Figure S9

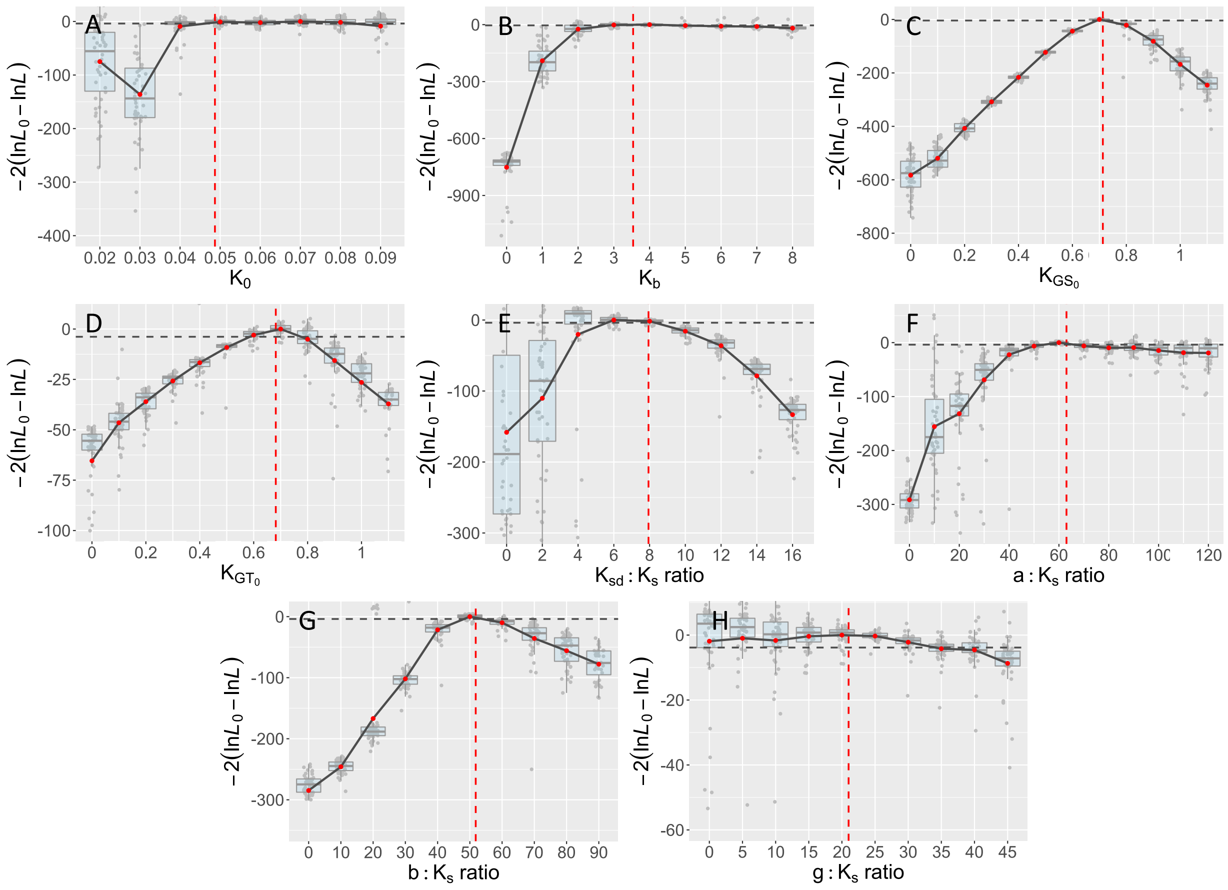

Supplementary Figure S10

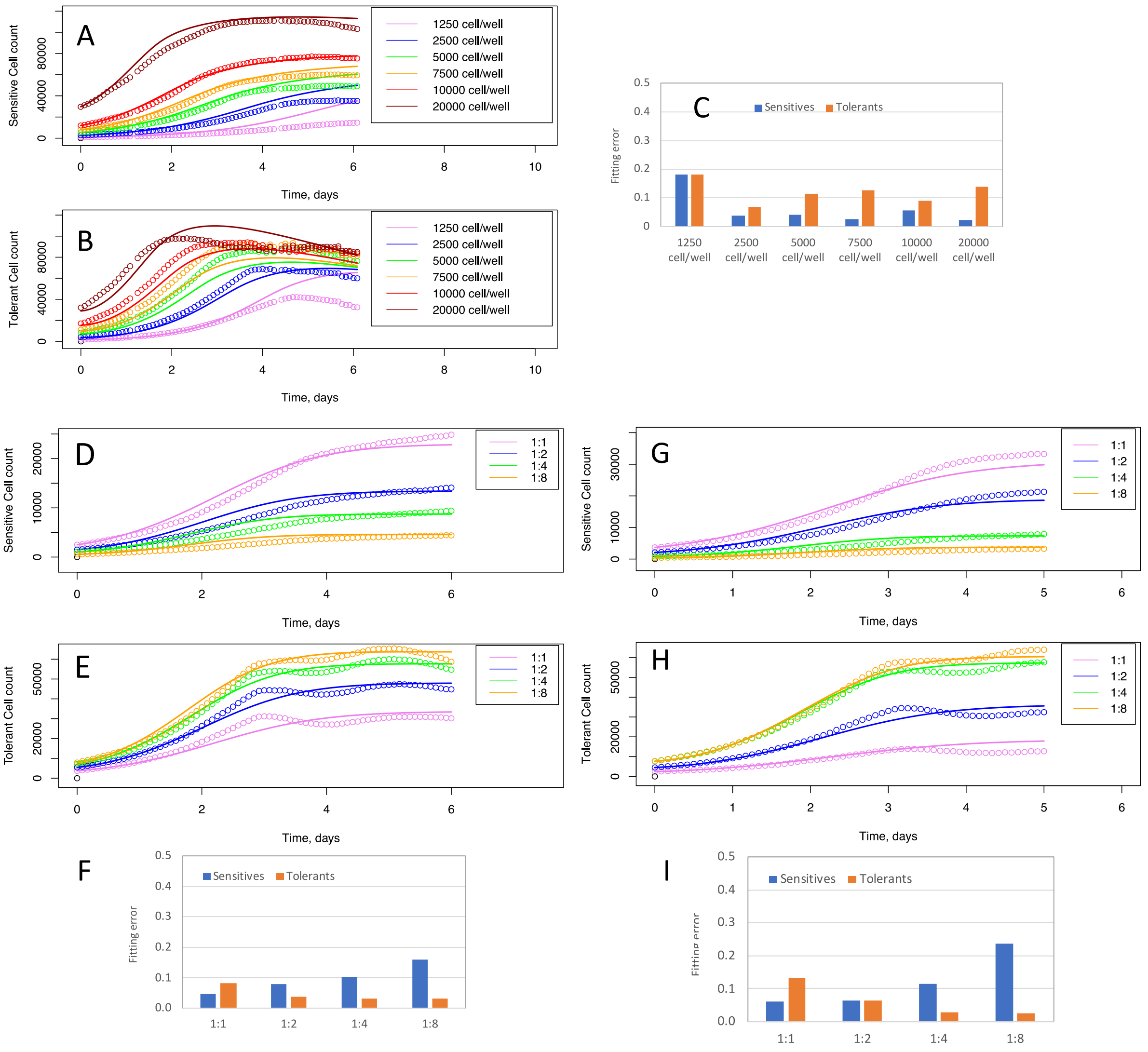

J

Condition	K_0	K_b	K_{Gs0}	K_{Gt0}	K_{str}	$K_{str,d}$	a	b	g
Sensitives only	9.51E-03	1.01	0.626	0.093	2.94E-04	6.06E-03	0.050	0.036	0.026
Tolerants only	1.00E-02	1.98	1.012	0.108	2.56E-04	2.70E-04	0.044	0.036	0.043
Heterotypic	4.87E-02	3.57	0.713	0.687	7.14E-04	5.64E-03	0.046	0.038	0.018
Heterotypic, 3 weeks	5.20E-02	2.60	0.708	0.189	6.74E-04	5.52E-03	0.050	0.038	0.020

Supplementary Figure S11

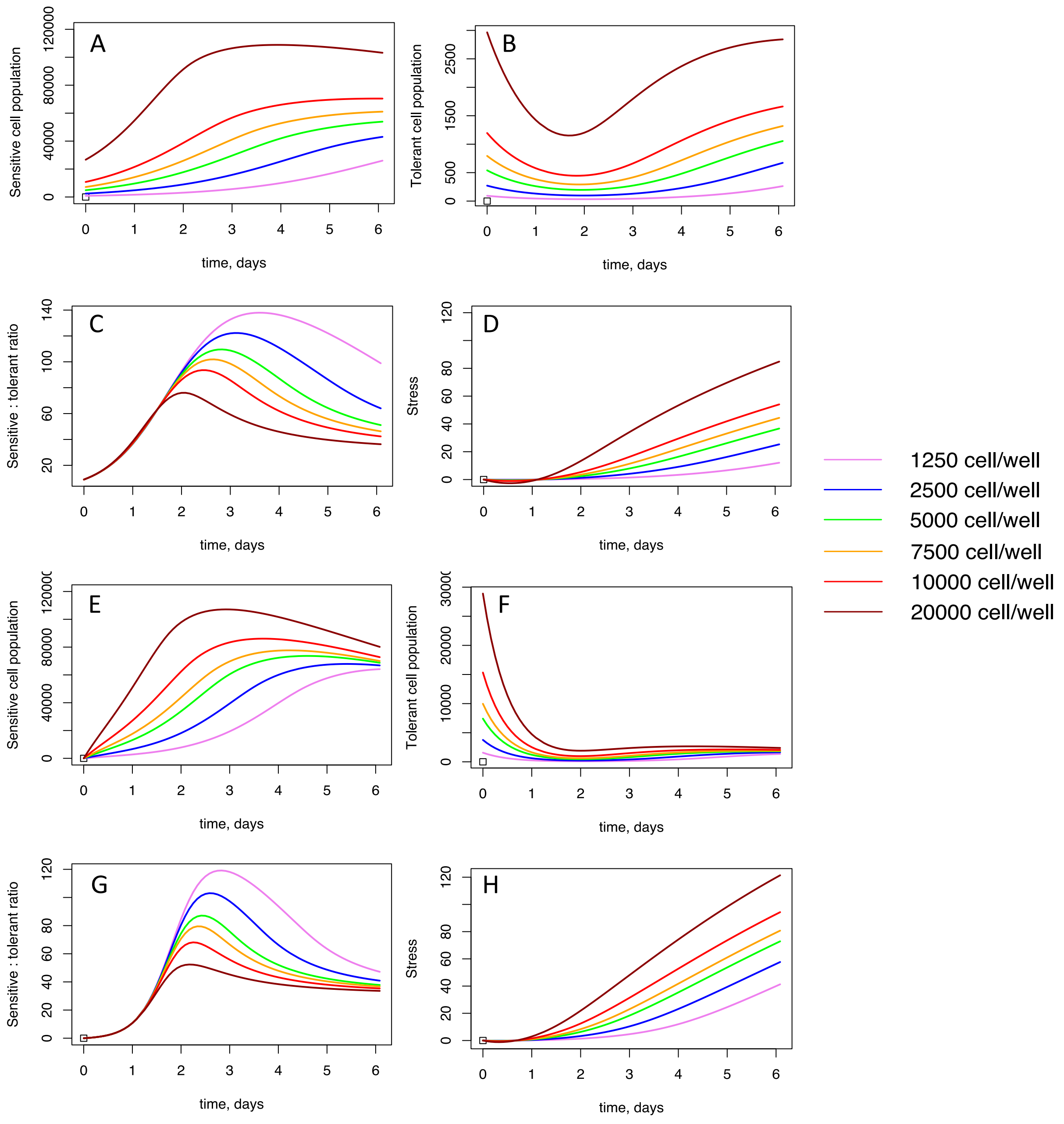

Supplementary Figure S12

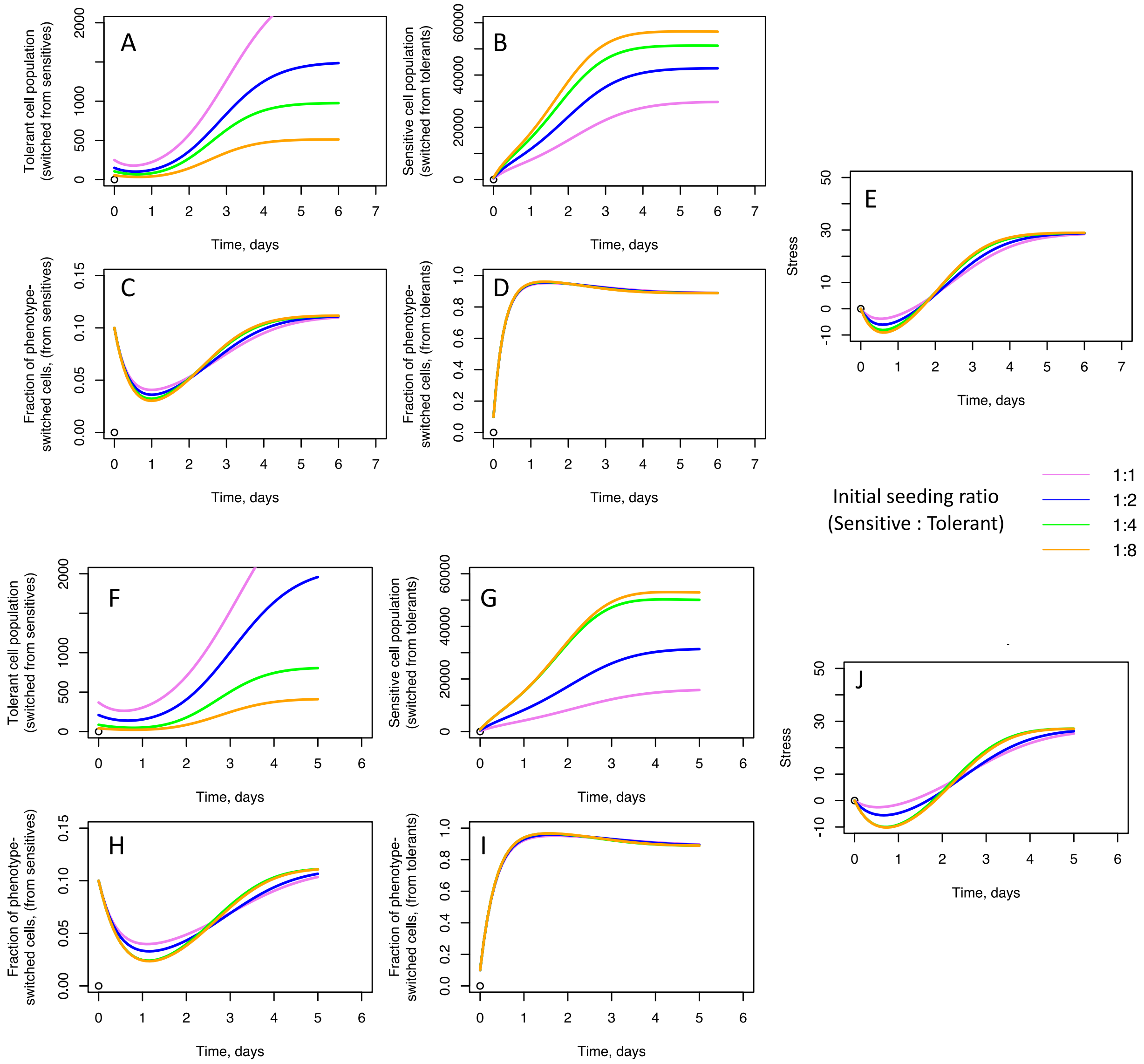

Supplementary Figure S13

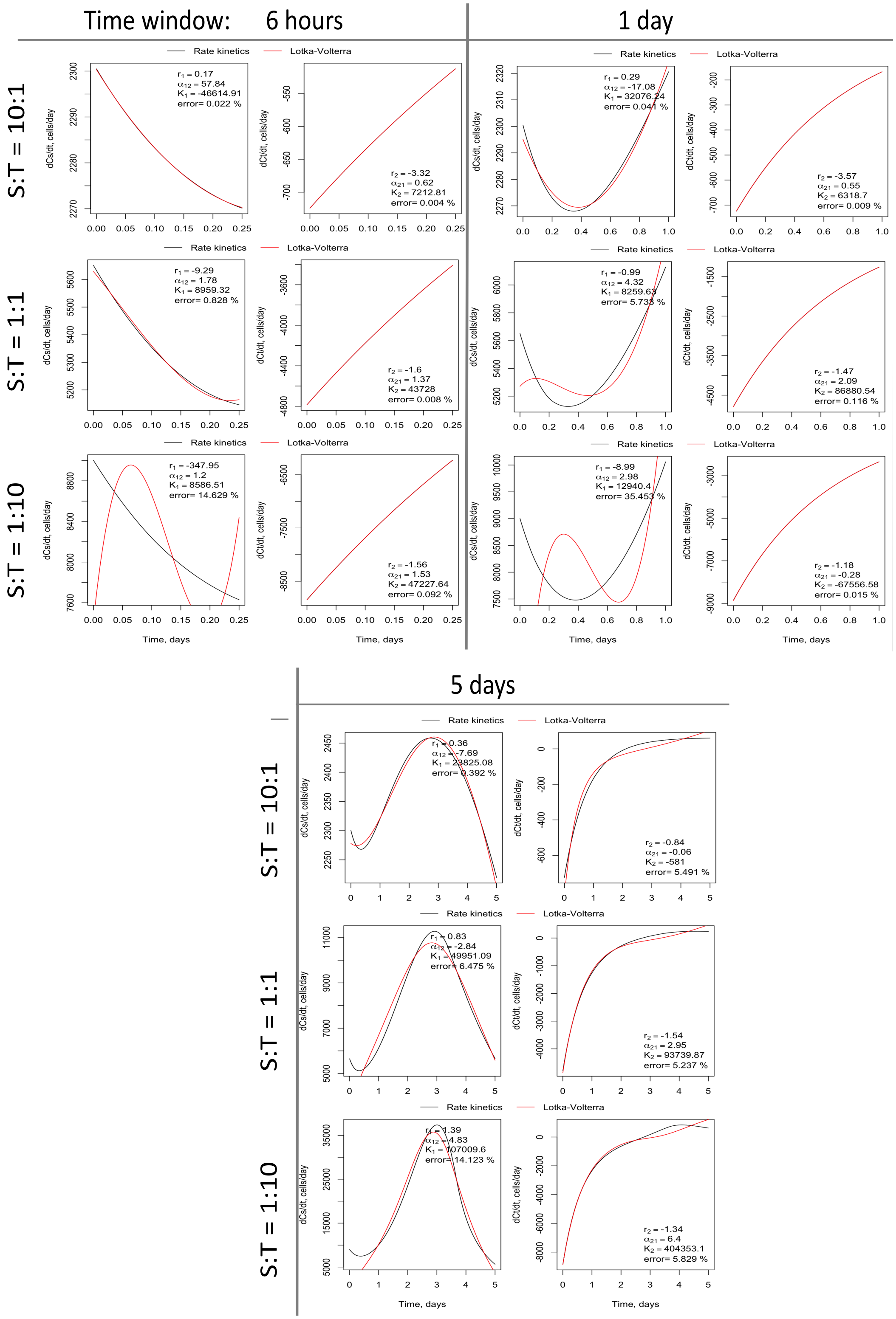

Supplementary Table S1. Changes in Tolerant: Sensitive ratios in co-culture

Row	Incubation period prior to live cell counting	Seeding ratio (Tolerant : Sensitive)	Ratio at first time point of live cell counting
1	12 hours	1:1	~2:1
2	12 hours	2:1	~4:1
3	12 hours	4:1	~7:1
4	12 hours	8:1	~13:1
5	3 weeks	1:1	~1:5
6	3 weeks	2:1	~2:1
7	3 weeks	4:1	~9:1
8	3 weeks	8:1	~15:1
9	3 weeks	1:1	~8:10
10	3 weeks	1:2	~2:10
11	3 weeks	1:4	~5:100
12	3 weeks	1:8	~5:100

Supplementary Table S2. Statistical significance for Figure S2C-E

Figure S2C				
T:S ratio	Time point	Statistical test	P-value	Significance
1:1	144 h	2-way ANOVA	<0.0001	Significant
2:1	144 h	2-way ANOVA	<0.0001	Significant
4:1	144 h	2-way ANOVA	<0.0001	Significant
8:1	144 h	2-way ANOVA	<0.006	Significant

Figure S2D				
T:S ratio	Time point	Statistical test	P-value	Significance
1:1	144 h	2-way ANOVA	0.5015	Not significant
2:1	144 h	2-way ANOVA	<0.0001	Significant
4:1	144 h	2-way ANOVA	<0.0001	Significant
8:1	144 h	2-way ANOVA	<0.006	Significant

Figure S2E				
T:S ratio	Time point	Statistical test	P-value	Significance
1:1	144 h	2-way ANOVA	<0.0001	Significant
2:1	144 h	2-way ANOVA	<0.0001	Significant
4:1	144 h	2-way ANOVA	<0.0001	Significant
8:1	144 h	2-way ANOVA	<0.0001	Significant

Supplementary Table S3. Statistical significance for Figure S2G

Figure S2G				
Experiment condition	Time point	Statistical test	P-value	Significance
Tolerant-conditioned media	72 h	2-way ANOVA	0.019	*
Sensitive-conditioned media	72 h	2-way ANOVA	0.001	***

Supplementary Table S4. Statistical significance for Figure S3A-C

Figure S3A-C			
S:T ratio	Statistical test	P-value	Significance
1:1	Wilcoxon two-tailed	<0.0001	Significant
2:1	Wilcoxon two-tailed	<0.0001	Significant
4:1	Wilcoxon two-tailed	<0.0001	Significant

Supplementary Table S5. Statistical significance for Supplementary Figure S2E-G

Supp. Figure S2E				
T:S ratio	Time point	Statistical test	P-value	Significance
1:1	144 h	2-way ANOVA	<0.0001	Significant
2:1	144 h	2-way ANOVA	<0.0001	Significant
4:1	144 h	2-way ANOVA	<0.0001	Significant
8:1	144 h	2-way ANOVA	0.1569	Not significant

Supp. Figure S2F				
T:S ratio	Time point	Statistical test	P-value	Significance
1:1	144 h	2-way ANOVA	<0.0001	Significant
2:1	144 h	2-way ANOVA	<0.0001	Significant
4:1	144 h	2-way ANOVA	<0.0001	Significant
8:1	144 h	2-way ANOVA	<0.006	Significant

Supp. Figure S2G				
T:S ratio	Time point	Statistical test	P-value	Significance
1:1	144 h	2-way ANOVA	<0.0001	Significant
2:1	144 h	2-way ANOVA	<0.0001	Significant
4:1	144 h	2-way ANOVA	<0.0001	Significant
8:1	144 h	2-way ANOVA	<0.0001	Significant

Supplementary Table S6. Statistical significance for Supplementary Figure S3E-G

Supp. Figure S3E				
S:T ratio	Time point	Statistical test	P-value	Significance
1:1	144 h	2-way ANOVA	<0.0001	Significant
2:1	144 h	2-way ANOVA	<0.0001	Significant
4:1	144 h	2-way ANOVA	<0.0001	Significant
8:1	144 h	2-way ANOVA	0.1569	Not significant

Supp. Figure S3F				
S:T ratio	Time point	Statistical test	P-value	Significance
1:1	144 h	2-way ANOVA	<0.0001	Significant
2:1	144 h	2-way ANOVA	<0.0001	Significant
4:1	144 h	2-way ANOVA	<0.0001	Significant
8:1	144 h	2-way ANOVA	<0.006	Significant

Supp. Figure S3G				
S:T ratio	Time point	Statistical test	P-value	Significance
1:1	144 h	2-way ANOVA	<0.0001	Significant
2:1	144 h	2-way ANOVA	<0.0001	Significant
4:1	144 h	2-way ANOVA	<0.0001	Significant
8:1	144 h	2-way ANOVA	0.3784	Not significant

Table S7. Fitted parameters from the Li et al.'s model

	K_S	K_T	γ_0	γ_1	δ_0	δ_1
Monitored immediately	65236	64219	1.3	-0.8	1.3	-1.0
Monitored after 3 weeks coculture	33266	63943	1.7	-0.8	1.3	-0.8

Table S8. Parameters for PSMSR using sigmoidal stress functions

	$C_{Str,K5}$	$C_{Str,K95}$	K_0	K_{max}	K_b	$C_{Str,GS5}$	$C_{Str,GS95}$	K_{GS0}	$K_{GS,min}$	$C_{Str,GT5}$	$C_{Str,GT95}$	K_{GT0}	$K_{GT,min}$	K_{Str}	$K_{Str,d}$
Monitored immediately	3.4	125.8	0.9	1.5	0.9	2.2	135.6	0.9	-0.6	3.1	153.8	0.8	-0.3	0.006	0.004
Three weeks coculture	2.1	143.5	0.8	1.4	0.9	2.4	132.3	1.0	-0.6	1.6	156.5	0.6	-0.4	0.006	0.005

Supplementary Figure Legends

Supplementary Figure S1. Generating heterotypic cultures in 1:1 ratio and increasing T:S ratios (2:1, 4:1, 8:1). (**A**) Cisplatin-sensitive cells (H23) and cisplatin-tolerant cells (H2009) stably express nuclear red fluorescent protein (RFP) and green fluorescent protein (GFP), respectively. (**B**) Images of cisplatin-sensitive cells (RFP) and cisplatin-tolerant cells (GFP) seeded in 1:1 ratio at timepoints 0 h and 72 h, in the untreated and cisplatin-treated (5 µM) condition. (**C**) Schematic representation of the experimental design of co-culturing sensitive (S) and tolerant (T) cells in T:S ratios of 1:1, 2:1, 4:1, and 8:1 for 3 weeks and collection of data points.

Supplementary Figure S2. Proliferative behavior of sensitive (S) and tolerant (T) cells mixed in increasing T:S seeding ratios 12 hours prior to the start of the experiment. (**A**) Schematic representation of the experimental design of mixing cells in various T:S ratios (1:1, 2:1, 4:1, and 8:1) 12 hours prior to cisplatin treatment and real-time monitoring of cell proliferation. (**B**) Proliferation rate of sensitive cells (red) and tolerant cells (green) in heterotypic culture over the course of 144 hours. (**C**) Proliferation rate of sensitive cells (purple) and tolerant cells (blue) in heterotypic culture treated with 5 µM cisplatin over the course of 144 hours. (**D**) Change in tolerant/sensitive cells ratio with (orange) and without (black) 5 µM cisplatin over the course of 144 hours. (**E**) Fold change in cell count after 144 h in untreated condition at various initial T:S seeding ratios (**F**) Fold change in cell count after 144 h in cisplatin-treated (5 µM) condition at various initial T:S seeding ratios (**G**) Ratio of T:S cells after 144 h in untreated and cisplatin-treated (5 µM) conditions. Two-way ANOVA was used for calculating statistical significance ****p<0.0001, **p=0.003, *p=0.04, ns-not significant. Statistical significance information can be found in **Supplementary Table 5**.

Supplementary Figure S3. Proliferative behavior of sensitive (S) and tolerant (T) cells grown in heterotypic cultures of increasing S:T seeding ratios for 3 weeks. (**A**) Schematic representation of the experimental design of co-culturing sensitive (S) and tolerant (T) cells in S:T ratios of 1:1, 2:1, 4:1, and 8:1 for 3 weeks and collection of data points. (**B**) Proliferation rate of sensitive cells (red) and tolerant cells (green) in heterotypic culture over the course of 144 hours. (**C**) Proliferation rate of sensitive cells (purple) and tolerant cells (blue) in heterotypic culture treated with 5 µM cisplatin over the course of 144 hours. (**D**) Change in tolerant/sensitive cells ratio with (orange) and without (black) 5 µM cisplatin over the course of 144 hours. (**E**) Fold change in cell count after 144 h in untreated condition at various initial S:T seeding ratios (**F**) Fold change in cell count after 144 h in cisplatin-treated (5 µM) condition at various initial S:T seeding ratios (**G**) Ratio of T:S cells after 144 h in untreated and cisplatin-treated (5 µM) conditions. Two-way ANOVA was used for calculating statistical significance ****p<0.0001, **p=0.001, *p=0.03, ns- not significant. Statistical significance information can be found in **Supplementary Table 6**.

Supplementary Figure S4. Schematic representation of the experimental design of continuous versus intermittent therapy in 2D cultures. Sensitive and tolerant cells were seeded in a ratio of 1:1, 2:1, and 4:1 in a 24-well plate and allowed to adhere overnight as depicted in the plate map. After 24 h (Day 1), the cells were treated with a sublethal dose (1 µM) of cisplatin for two days. After 2 days (Day 3), all cells were passaged 1:5. The "Continuous" group received fresh media containing 1 µM cisplatin. The "Intermittent" group received fresh medium only (no cisplatin). Cells were allowed to grow for 10 days and proliferation was monitored in real-time using IncuCyte. After 10 days (Day 13), cells were trypsinized. The "intermittent" group was split into 2 groups: "Intermittent – 1 cycle" and "Intermittent – 2 cycles". All cells were passaged 1:5 and re- seeded in a 24-well plate as depicted in the plate map. After 24 h (Day 14), the "Continuous" group and "Intermittent – 2 cycles" group were treated with 1 µM cisplatin. Only fresh media was added to the "Intermittent – 1 cycle" group. Cell proliferation was monitored in real-time for 4 days using IncuCyte. After 4 days (Day 18), cisplatin was removed, and fresh media was added to

"Intermittent – 2 cycles" group. Fresh media was added to "Intermittent – 1 cycle group" and fresh media containing 1 µM cisplatin was added to "Continuous" group. Cell proliferation was monitored in real-time for 25 days using the IncuCyte Live Cell Imaging System. The experiment was terminated after 25 days (Day 43).

Supplementary Figure S5. Comparison between PSMSR and the model by Li et al. (A, C) fitting of the Li et al model to the sensitive and tolerant cell growth trends respectively, where the cells were mixed and monitored immediately, (B, D) corresponding graphs where the cells were cocultured together for three weeks prior to monitoring their growth, (E, G) fitting of PSMSR where the cellular growth was monitored immediately after mixing, (F, H) fitting of PSMSR where the cellular growth was monitored after three weeks of coculture, (I, J) fitting errors in the sensitive cell growth trends compared between the PSMSR and the Li et al model where the cells were (I) monitored immediately after mixing and (J) after three weeks of coculture, (K, L) fitting errors for the tolerant cells when (K) monitored immediately after mixing and (L) after three weeks of coculture.

Supplementary Figure S6. Sigmoidal stress relationships modeled in PSMSR. The parameters describing the sigmoidal functions are highlighted in the figures (A) variation of phenotypic switching equilibrium constant with stress, (B) variation of sensitive cell growth rate with stress, (C) variation of tolerant cell growth rate with stress.

Supplementary Figure S7. Fitting of PSMSR with sigmoidal growth functions. Lines represent the fitted trends, while circles represent the experimentally measured populations. (A, C) Sensitive and tolerant cell growth with time where cells were monitored immediately after mixing, (B, D) Sensitive and tolerant cell growth with time where cells were monitored after three weeks of coculture, (E,F) Comparison of fitting errors between PSMSR models with linear and sigmoidal growth functions.

Supplementary Figure S8. Profile likelihood analysis for PSMSR parameters for the experiments where the cells were monitored immediately after mixing. This figure shows the zoomed-in views of the significance regions. For original plots, please see Fig. S9. $\ln L$ represents the log- likelihood and $\ln L_0$ the maximum mean log-likelihood within the range of tested parameter values. In each panel, the name of the parameter is given below the plot. The likelihood for a given parameter was obtained by fixing the parameter through different values and optimizing the rest of the parameters by running the GA optimization 50 times each. The boxes represent the interquartile ranges, and the log-likelihood values from individual optimizations are shown as grey points (with added jitter for easy identification). The red points are the mean log-likelihood values. The dashed horizontal line represents the lower threshold for significance based on 95% confidence (95% quantile of the χ^2 distribution with one degree of freedom). The vertical dashed lines represent the parameter values that were used in the analysis throughout the manuscript. When the red dashed line intersects the mean profile log-likelihood curve above the significance threshold, it indicates that the chosen parameter is significant in identifying the model from the experimental data. The significant range for each parameter is shown in blue font. For parameters $K_{Str,d}$, a, b and g (E-H), ratios with K_s are analyzed.

Supplementary Figure S9. Profile log-likelihood functions for PSMSR parameters for the experiments where the cells were monitored immediately after mixing. This figure shows the original plots, while Fig. S8 shows the zoomed-in views of the significance regions.

Supplementary Figure S10. Fitting of the PSMSR model to the cellular growth curves. (**A**) Sensitive cells only; (**B**) tolerant cells only; the curves are colored according to the initial seeding population; circles represent the cell populations at different time points as means of three replicates; lines represent the fitted trends; (**C**) estimated fitting error for sensitive only (blue) and tolerant only (orange) populations; (**D, E**) sensitive and tolerant cell populations, where the cells were mixed at different proportions and counting was started immediately; the colors represent the growth curves from different initial seeding proportions, as indicated in the legend (sensitive to tolerant cell seeding ratios); (**F**) fitting error for sensitive and tolerant cell growth in the mixed culture; (**G-I**) equivalent results for the mixed cell populations, where the cells were cultured together for three weeks before monitoring growth; (**J**) parameter values for the various cell systems; the parameters that significantly differed for the 3 weeks cultured population compared to the other systems are highlighted in green (increase) or red (decrease).

Supplementary Figure S11. Predicted evolution of phenotypic switching and stress in systems seeded with sensitive or tolerant cells alone. (**A-B**) Populations of sensitive and (switched) tolerant phenotypes with time, when seeded with sensitive cells only; (**C-D**) ratio of sensitive to tolerant phenotype (C) and stress (D) as functions of time; (**E-F**) populations of (switched) sensitive and tolerant phenotypes when seeded with tolerant cells only; (**G-H**) ratio of sensitive to tolerant phenotype (G) and stress (H) as functions of time; colors represent different seeding populations.

Supplementary Figure S12. Predicted evolution of switched phenotypes and stress in mixed culture experiments. **A-E** represent systems where growth was monitored immediately after mixing; **F-J** represent systems where growth was monitored three weeks after mixing; (A, F) tolerant cell population with time that have switched their phenotype from sensitive cells; (B, G) sensitive cell population with time that have switched phenotype from tolerant cells; (C, H) fraction of sensitive cells that have switched to the tolerant phenotype, as function of time; (D, I) fraction of tolerant cells that have switched to the sensitive phenotype, as function of time; (E, J) stress with time; colors are according to the initial seeding ratio of sensitive to tolerant cells as shown in the legend; the total cell population in each case was close to 5000.

Supplementary Figure S13. Integration between the PSMSR and competitive Lotka-Volterra models. The Lotka-Volterra model parameters are derived by fitting the LV equation to the growth rate of sensitive and tolerant cells as calculated by the PSMSR model over different time windows, initial seeding of 7500 total cells with different sensitive to tolerant mixing ratios; the graphs show the comparison between growth rates calculated from both models; rates from PSMSR model are in black; rates from LV equation fitting are in red; the best fitted parameter values and fitting error are shown in the legend of each plot.
