# Supplementary material for: Dynamic Phenotypic Switching and Group Behavior Help Non-Small Cell Lung Cancer Cells Evade Chemotherapy
Source: Biomolecules. 2021 Dec 21;12(1):8. doi: 10.3390/biom12010008 (PMC8773639; doi:10.3390/biom12010008)
Supplement: Supplementary file 1 [file biomolecules-12-00008-s001.zip › Supplementary_document Final.pdf]

Supplementary Text

Section S1: Comparison of the Li et al model to PSMSR

We have compared the model by Li et al.[1] to the PSMSR by fitting both the models to the cell growth data. The mathematical form of Li et al.'s model is as follows:

$$\frac{dS}{dt} = \left(r_S - \frac{r_S}{K_S}S - \frac{r_T}{K_S}T\right)S \quad (1)$$

$$\frac{dT}{dt} = \left(r_T - \frac{r_T}{K_T}T - \frac{r_S}{K_T}S\right)T \quad (2)$$

, where S,T are sensitive and tolerant cell populations, r_S, r_T are sensitive and tolerant cell growth rates and K_S, K_T are the carrying capacities. Both r_S and r_T are linear functions of the sensitive cell frequency:

$$r_S(x) = \gamma_0 + \gamma_1 x \quad (3)$$
$$r_T(x) = \delta_0 + \delta_1 x \quad (4)$$

, where $x = \frac{S}{S+T}$, and $K_S, K_T, \gamma_0, \gamma_1, \delta_0, \delta_1$ are constant parameters, to be determined by fitting to the experimental data.

Due to the relatively simplistic form of equations 1-2, nonlinear optimization (using the method L-BFGS-B [2]) rather than Generic Algorithm was used for the fitting. The initial guesses for $\gamma_0, \gamma_1, \delta_0, \delta_1$ were obtained as follows. First, the initial growth rates were estimated by fitting an exponential model to the initial phase of the growth trends (t = 0-2 days). The growth rates obtained from the various S:T ratios were then fitted to a linear model to obtain $\gamma_0, \gamma_1, \delta_0, \delta_1$. These values were used as initial guesses in the nonlinear optimization. The initial guesses for the carrying capacities were set to the maximum values of the sensitive and tolerant cell populations among all the experiments. As with every other model in this manuscript, the fitting was performed separately for the 'monitored immediately' and 'monitored, three weeks' experiments. Convergence was achieved when the error tolerance became less than 10^{-8}. The fitted parameter values are given in **Supplementary Table S7**. Comparison between the PSMSR and the Li et al.'s model suggests that the two models fit the data with similar accuracy only for the sensitive cell populations where the cells were monitored immediately after mixing (**Supplementary Figure S5A & I**). For all other cases, the PSMSR gives lower fitting error compared to Li et al.'s model. Especially for the sensitive cell data from the 'monitored, three weeks' experiments, Li et al.'s model shows significant deviation from the observed trends (**Supplementary Figure S5B**), as compared to PSMSR (**Supplementary Figure S5F**).

Section S2: Details on PSMSR

Significance of stress in affecting the cellular growth

The most striking premise in the PSMSR model is the involvement of stress in mediating phenotype switching between the sensitive and the tolerant cells, which is supported by our recent work[3]. We used fluorescence-activated cell sorting (FACS) to sort the tolerant cells based on expression of integrin beta 4 (ITGB4), a biomarker of cisplatin resistance. After 4 days, low ITGB4-expressing tolerant cells (cisplatin-sensitive phenotype) stochastically switched to express higher ITGB4 (cisplatin-tolerant phenotype). Further, this switching to the tolerant phenotype was enhanced by the administration of cisplatin, thus indicating the relevance of stress-mediated phenotype switching. In PSMSR, we assume that at any time, a fraction of one cellular phenotype (e.g. sensitive) transforms to another phenotype (e.g. tolerant), and that the equilibrium between these two phenotypes shifts with environmental stress. Although in our work, we do not directly measure stress level, influence of environmental stress on cellular behavior is well documented in the cancer literature.[4,5] Briefly, we envision stress as a collection of factors in the cellular microenvironment that inhibits growth and increases cell death (schematic in **Figure 4A**). Such factors can include metabolic byproducts, growth inhibitory proteins, free radicals, lack of oxygen and nutrients, all of which collectively impact cell survival.

Explanation of PSMSR parameters

In the PSMSR model equations 1 and 2, K_a and K_b are the rate of switching from sensitive to the tolerant phenotype and vice versa.[6] We assume K_a to be linearly dependent on stress and K_b to be fixed. This assumption is in agreement with the fact that the switching from the sensitive to the tolerant phenotype is mediated through upregulation of transcription through histone deacetylation and is more amenable to modulation via stress or HDAC inhibitors such as suberoylanilide hydroxamic acid (SAHA) that interfere with chromatin remodeling.[7] However, the reverse switching is not known to be affected by such factors.

The relationships between K, K_{GS}, K_{GT} and stress are modeled as linear functions below a threshold stress C^t_{Str}, and as piecewise continuous asymptotic functions beyond this threshold.

$$K(C_{Str}) = \begin{cases} K_0(1 + aC_{Str}), & C_{Str} \leq C^t_{Str} \\ K_0(1 + aC_{Str}) + (K_{max} - K_0)\left[\frac{a_1 C_{Str}}{1 + a_1 C_{Str}} - \frac{1}{2}\right], & C_{Str} > C^t_{Str} \end{cases} \tag{5}$$

$$\text{Here, } C^t_{Str} = \frac{1}{4a}\left[\frac{K_{max}}{K_0} - 1\right],\ a_1 = {C^t_{Str}}^{-1} \tag{6}$$

$$K_{GS}(C_{Str}) = \begin{cases} K_{GS_0}(1 - bC_{Str}), & C_{Str} \leq C_{Str}^t \\ K_{GS_0}\left(1 - 2bC_{Str}^t + b\frac{{C_{Str}^t}^2}{C_{Str}}\right), & C_{Str} > C_{Str}^t \end{cases} \tag{7}$$

$$K_{GT}(C_{Str}) = \begin{cases} K_{GT_0}(1 - gC_{Str}), & C_{Str} \leq C_{Str}^t \\ K_{GT_0}\left(1 - 2gC_{Str}^t + g\frac{{C_{Str}^t}^2}{C_{Str}}\right), & C_{Str} > C_{Str}^t \end{cases} \tag{8}$$

where K_{GS_0} and K_{GT_0} are growth rates in absence of stress and *b, g* represent the sensitivity of the growth rates to stress variation. We assume that the equilibrium constant for phenotype switching increases linearly with stress, becoming asymptotic at high stress, approaching a plateau at K_{max}. To mimic the asymptotic behavior, the trend switches from linear to sigmoidal beyond a threshold stress given by C_{Str}^t. The value of C_{Str}^t is chosen to ensure continuity in the first derivative of equation 5. The choice of a linear function was to establish a monotonic relation between stress and phenotypic switching, and is motivated by experimental observations that stress leads to up regulation of phenotypic switching in cancer cells [8]. However, experimental data to identify an exact quantitative relationship between stress and phenotypic switching is not yet available. Hence, we assumed a simple linear relation between the two. Notably, the cellular growth model can be fitted to the experimental data using a different monotonic relation such as a sigmoidal function **(Supplementary text, Section S3**). However, this would increase the number of parameters and make the fitting more challenging without providing any additional insights and was therefore not considered. Likewise, the cellular growth rates K_{GS} and K_{GT} decrease linearly with stress, before becoming asymptotic at high stress, reaching the values $K_{GS_0}(1 - 2bC_{Str}^t)$ and $K_{GT_0}(1 - 2gC_{Str}^t)$ respectively.

Significance of 'AUC'

In cancer pharmacology, AUC is a widely used metric in many different applications, especially in estimating drug toxicity among different doses and regimens. However, AUC-dependent death rate has also been used in kinetic models describing cancer cell populations over time [9]. It is noteworthy that the current model does not take into account some of the kinetic effects associated with AUC-based models, such as diffusion of drug in to and out of the cells and decay of drug over time. Moreover, cisplatin can produce bystander effect, where the growth of nearby healthy cells can be affected by soluble factors released by stressed cells[10]. We have not incorporated these effects in the current model, which focuses on understanding group behavior, rather than precise quantitative effect of cisplatin. The simpler AUC based model that we have used agreed reasonably well with the experimental trends.

Details of intermittent therapy simulations

The intermittent therapy simulations were performed using the PSMSR model (equations 5-6, main text), following the experimental drug regimens as explained in **Supplementary Figure S4**. The parameters which were used correspond to the ones obtained by fitting the PSMSR model to the co-culture growth data in presence of cisplatin, where the cells were mixed and counted after 12 hours. The cell passage was implemented by resetting the stress to zero and reducing the cell population accordingly. During the intermittent phase where cisplatin is withdrawn, the AUC-dependent effect is still maintained based on the initial exposure of the cells to cisplatin. A more sophisticated model would have been one where the AUC effect is slowly decayed over time when cisplatin is removed. However, in our case, the memory effect of cisplatin on cellular death is maintained unchanged throughout the remainder of the experiment. In intermittent therapy, the cisplatin exposure to the cells was for a limited duration (two days), hence its effect is not as drastic as in continuous therapy. Nevertheless, this is a potential limitation of the model.

Fitting of the phenotype-switch model to experimental growth data

According to the original premise of PSMSR, in each co-culture, a fraction of the GFP tagged sensitive population will exist as phenotypically tolerant. Likewise, a fraction of the RFP tagged tolerant population will be phenotypically sensitive. Our growth recording setup cannot distinguish between these subpopulations, which can only measure growth based on whether the cells are green or red fluorescent. Therefore, for fitting purpose, we model the GFP tagged cell population as $P_{GFP} = S_{GFP} + T_{GFP}$ (where P_{GFP} is the total GFP tagged cell population, S_{GFP} represents the GFP tagged phenotypically sensitive subpopulation and so on). Likewise, the RFP tagged cell population can be expressed as $P_{RFP} = S_{RFP} + T_{RFP}$. These four subpopulations are modeled using the PSMSR equations 1, 2 and 4, leading to five coupled ordinary differential equations (two for the sensitive subpopulations, two for tolerant subpopulations and one for stress). These ODEs are solved numerically to obtain the cellular subpopulations as function of time. For initial conditions, T_{GFP} is set to 10% of P_{GFP} and S_{RFP} is set to 10% of P_{RFP}, based on our earlier work [3]. The fitting of the equations to the experimental growth data starts with a set of initial guesses for the parameters, as discussed in the next paragraph. At each iteration of the parameter optimization, the ODEs are solved numerically using the current parameter values and the resulting P_{GFP} and P_{RFP} for all time points are compared to the corresponding experimental values to calculate the root mean square error (RMSE), which is used as the objective function for the optimization. The numerical solution of the ODEs was obtained using the LSODA method [11] as implemented in the R package deSolve [11,12].

The fitting was performed using the global optimization method, Genetic Algorithm (GA) [13], as implemented in the package GA in R.[14] First, a local optimization

using the Nelder-Mead nonlinear optimization was performed for each growth trend individually. The fitted parameters that showed reasonable agreement with experiments were used to estimate the range of variation for each parameter and were provided as lower and upper bounds to the GA routine. In GA, the population size was set to 50 and the maximum number of iterations was 5000. All other settings were kept at their default values. The fitting started with an initial guess for each parameter that was obtained by averaging the corresponding parameter values obtained from the Nelder-Mead optimization. The optimization was considered to have converged once the best solution did not change for more than 100 steps. To test the robustness of the GA optimization, we performed the optimization 100 times and used the resulting spread in parameter values to estimate the error and 95% confidence limits of the derived parameters according to the student's t distribution. All parameter values were associated with reasonably low error, as seen from the 95% confidence limits in **Table 1**, suggesting that the optimization was robust. The fitting was performed separately using the growth data collected from the sensitive and the tolerant cells cultured as monotypic or heterotypic co-cultures, leading to separate parameter sets for each of these experiments. Notably, we fitted these experimental data separately because the co-culture of sensitive and tolerant cells appeared to alter their phenotypic behavior. Therefore, the monotypic and the heterotypic experiments must be treated as separate systems with their own model parameters. By comparing the model parameters across the different cultures (e,g, monotypic vs. heterotypic or monitored immediately after mixing vs. mixed for three weeks), we can understand which cellular parameters were significantly affected due to the change of culture conditions. The fitting error was estimated as the mean of the absolute deviations between the observed and the fitted trends, normalized by the cell population range.

Given the complexity of the PSMSR model and the relatively large number of model parameters, we wanted to assess whether the functional form of the model is uniquely identifiable from our experimental data. This question was addressed by calculating the log-likelihood profiles for the PSMSR parameters near their optimal values, the details of which can be found in the **Supplementary text**, **Section S4** and **Supplementary Figures 8 and 9**). From this analysis, we found that the majority of the parameters showed narrow ranges of significant values around the optima indicating that the dynamic growth trends can be uniquely captured by PSMSR with reasonable confidence.

Figure 4B and **4C** and **Supplementary Figure S10** show the agreement between the fitted and observed growth trends for different types of systems (monotypic sensitive or tolerant cell cultures, heterotypic cultures where counting begun immediately or after three weeks of co-culture). For each system, a single set of parameters fitted the different growth curves (various seeding populations or mixing proportions) with reasonable

agreement (please see the fitting errors in **Supplementary Figure S10C, 10F** and **10I** and parameter values in **Table 1**). Among the sensitive or tolerant only systems, the worst error was observed for the lowest seeding population (**Supplementary Figure S10C**, 1250 cells/well). It is possible that at low seeding populations, stochastic effects would dominate, and the systems may be best modeled using stochastic differential equations instead of deterministic ones. In the mixed system where cell counting was performed immediately, the error increased proportionally with the number of tolerant cells in the system, indicating that the tolerant cell behavior may be more complex than what the model describes. The errors were relatively low for the systems after three weeks of co-culture, since the majority of the tolerant cells were predicted to have switched their phenotypes to be sensitive after three weeks, as will be discussed later. Hence, the growth dynamics after three weeks was dominated by the sensitive phenotype, even when seeded with high population of green fluorescent cells that were descended from tolerant ancestors.

Section S3: PSMSR using sigmoidal stress relationship

In this section, we address the question whether the agreement of the PSMSR model with experiments is sensitive to the choice of the stress-growth rate (or K_a) relationship. In the model presented in this manuscript, we have used linear functions with asymptotic behavior at high stress. We also fit the PSMSR model to the experimental data using sigmoidal functions of the following form:

$$K(C_{Str}) = K_0 + \frac{(K_{max} - K_0)}{1 + exp\left[ln19\left(1 - 2\frac{C_{Str} - C_{Str,K5}}{C_{Str,K95} - C_{Str,K5}}\right)\right]} \tag{9}$$

$$K_{GS}(C_{Str}) = K_{GS_0} - \frac{\left(K_{GS_0} - K_{GS_{min}}\right)}{1 + exp\left[ln19\left(1 - 2\frac{C_{Str} - C_{Str,GS5}}{C_{Str,GS95} - C_{Str,GS5}}\right)\right]} \tag{10}$$

$$K_{GT}(C_{Str}) = K_{GT_0} - \frac{\left(K_{GT_0} - K_{GT_{min}}\right)}{1 + exp\left[ln19\left(1 - 2\frac{C_{Str} - C_{Str,GT5}}{C_{Str,GT95} - C_{Str,GT5}}\right)\right]} \tag{11}$$

, where K_0, K_{max}, $C_{Str,K5}$, $C_{Str,K95}$, K_{GS_0}, $K_{GS_{min}}$, $C_{Str,GS5}$, $C_{Str,GS95}$, K_{GT_0}, $K_{GT_{min}}$, $C_{Str,GT5}$, $C_{Str,GT95}$ are constant parameters to be determined from fitting to the experimental growth trends. The physical meaning of these parameters are as follows; K_{max} is the maximum achievable phenotype switching equilibrium constant and K_0 is the lowest equilibrium constant at zero stress. $C_{Str,K5}$ and $C_{Str,K95}$ are the stress levels at which the equilibrium constant values

show 5% and 95% increase relative to *K0*. Likewise, K_{GS_0}represents the growth rate of sensitive cells at zero stress and $K_{GS_{min}}$represents the lowest growth rate at infinite stress. *CStr,GS5* and *CStr,GS95* are the stress levels at which the growth rates show 5% and 95% decrease relative to K_{GS_0}. Similar interpretation applies to the tolerant cell growth function. The functional forms of these variables are shown in **Supplementary Figure S6**. The fitting was performed using Genetic Algorithm following the same protocol as described in the main manuscript. **Supplementary Figure S7** shows the comparison of the fitted trends to the experimental observations for cases where the cells were monitored immediately, as well as after three weeks of co-culture (optimal parameter values can be found in **supplementary Table S8**). Compared to the PSMSR model using linear growth functions (**Supplementary Figure S5E–H**), using sigmoidal growth functions gives similar levels of agreement with the experimental trends (although sensitive cells at S:T seeding ratio of 1:8 and tolerant cells at seeding ratio of 1:1 show somewhat higher fitting error while using the sigmoidal functions), as shown by the fitting errors (**Supplementary Figure S7E,F**). This suggests that the choice of the stress functional form is less important to the model, as long as a monotonic relation is maintained. However, to come to a definitive conclusion, one has to test several different monotonic functions, which is beyond the scope of the current work.

Section S4: Profile likelihood of PSMSR parameters

In this section, we address the issue, whether the functional form of the PSMSR is uniquely identifiable from the experimental data. To this end, we calculate the profile log-likelihood function ln*L* for each PSMSR parameter over a limited range near its optimal value. To calculate ln*L* for a given parameter θ, we hold θ fixed at different values and at each step, optimize the rest of the parameters using GA. Assuming a gaussian error model, the profile log-likelihood is then given by the following equation[15,16]:

$$\ln[L(\theta|Y)] = -\frac{n}{2}\ln(2\pi\sigma^2) - \frac{SSQ(\theta;Y)}{2\sigma^2} \qquad 12$$

where, Y is the experimental dataset, n is the number of datapoints (240 in our case), σ is the measurement error and SSQ (sum of squares) = $\sum_{i=1}^{n}(Y - Y_{pred})^2$. By substituting the maximum likelihood estimate (MLE) estimate of $\sigma^2 = \frac{SSQ(\theta;Y)}{n}$ in equation 12, we obtain,

$$\ln[L(\theta|Y)] = -\frac{n}{2}\ln SSQ(\theta;Y) + C \qquad 13$$

where C is a constant. Calculating ln*L* thus requires optimizing the rest of the parameters for every value of θ. This is challenging with GA, since running the optimization each time can give a slightly different answer. We therefore performed the optimization 50 times for each θ (400-500 GA optimizations per parameter), and calculated the mean ln*L*, as shown in **Supplementary Figure S9**. For sufficiently large number of datapoints, the significance threshold corresponding to 95% confidence is given by[15,16]:

$$2(lnL_0 - lnL) \leq icdf(\chi_1^2, 0.95) \quad 14$$

, where L_0 is the likelihood when all parameters are set to their optimal values, and *icdf* is the inverse cumulative density function of the χ^2 distribution with one degree of freedom.

Supplementary Figure S8 shows the zoomed in views of the profile plots to better highlight the significant ranges of each parameter (the original plots are given in the supplementary material). According to the PSMSR model equations, the scaling of the parameters K_s, $K_{Str,d}$, a, b and g will change according to the scale of the stress variable C_{Str}. Since C_{Str} is a hidden variable in the model, its scale is arbitrary and may vary among the individual optimizations. To address this, we calculated the likelihoods for the ratios of $K_{Str,d}$, a, b and g to K_s, which are unaffected by the scaling of C_{Str}.

From **Supplementary Figure S8** , many of the parameters show narrow ranges of significant values indicating, that these parameters are strongly sensitive to the experimental data. The parameter K_0 (**Supplementary Figure S8**A) shows a relatively wide range of significant values (0.045 – 0.085), whereas the parameter g (**Supplementary Figure S9H**) is mostly insensitive to the accuracy of the model fitting (significant range < 35 g:K_s). The parameter g relates the tolerant cell growth rate to stress. Interestingly, based on our knowledge of cellular behavior, the tolerant cell growth is expected to be less affected by stress compared to the sensitive cells. This insensitivity to stress could be reflected in the behavior of the parameter g. Considering the narrow significant ranges observed in the profile likelihoods of majority of the parameters, we can conclude that the functional form of the PSMSR can be uniquely identified from the cellular growth data.

Section S5: Epigenetic modulation can distinguish drug sensitivity, tolerance and resistance in lung cancer

To test the possibility that drug sensitivity can be regulated at the epigenetic level in a reversible way, as opposed to genetic mutations alone, we used two different epigenetic modulators namely, 5-azacytidine (5-AZA), a DNA methyltransferase

inhibitor, and suberoylanilide hydroxamic acid (SAHA), a histone deacetylase inhibitor, and determined their effects on cisplatin resistance.

While SAHA treatment did not enhance the effect of cisplatin on sensitive H23 cells (**Figure 3H**) or H1993 cells that are resistant to cisplatin (IC50>300µM) (**Figure 3J**), it had a significant additive effect on the H2009 cells, suggesting that these cells can become sensitive through epigenetic intervention (**Figure 3I**). However, 5-AZA had no discernable effect (not shown), suggesting that epigenetic regulation of chromatin rather than specific cytosine residues in the DNA modulates cisplatin tolerance in the H2009 cells. Based on these criteria, H2009 qualify as cisplatin-tolerant (reversible) rather than resistant (irreversible) while H1993 may represent a truly resistant phenotype. Taken together, these observations suggest that tolerance to cisplatin can be reversed unless the tolerant cells acquire mutations making them irreversibly resistant.

Section S6: Experimental Materials and Methods

Cell lines and reagents. Cell lines (H23, H2009, H1993) were obtained from American Type Culture Collection (ATCC) (Manassas, VA, USA) and cultured in RPMI 1640 medium (Corning) supplemented with 10% fetal bovine serum (FBS), L-glutamine (2 mM), penicillin/streptomycin (50 U/ml), sodium pyruvate (1 mM), and sodium bicarbonate (0.075%) at 37°C, 5% CO_2. Cisplatin was provided by City of Hope National Medical Center clinics (Duarte, CA, USA). Puromycin was purchased from Thermo Fisher Scientific (Waltham, MA, USA). Suberoylanilide hydroxamic acid (SAHA) was purchased from Selleck Chemicals (Houston, TX, USA).

Live cell imaging and analysis. Cell lines H23 and H1993 were stably transfected with NucLight Red Lentivirus (Essen Bioscience, Ann Arbor, MI, USA) to express nuclear mKate2, a red fluorescent protein (RFP), and H2009 was stably transfected with NucLight Green Lentivirus to express nuclear green fluorescent protein (GFP). Stable clones were selected with puromycin (1 µg/ml). Live cell images were acquired in real time using the IncuCyte Live Cell Imaging System (Essen Bioscience). Cell counting masks were generated using the IncuCyte software to perform data analysis.[3]

Monotypic/heterotypic culture cell proliferation and drug sensitivity assay. For 1:1 heterotypic cultures 2.5×10^5 H23 (cisplatin-sensitive) cells and 2.5×10^5 H2009 (cisplatin-tolerant) cells (5×105 total cells) were plated on a 10 cm dish. For heterotypic cultures with different sensitive: tolerant seeding ratios (1:2, 1:4, 1:8, 2:1, 4:1, 8:1), numbers of each cell type were adjusted accordingly and plated with 5×10^5 total cells per plate. Cultures were

maintained in 37°C, 5% CO_2 for 3 weeks and passaged 1:5 when confluent (every 4-5 days). After 3 weeks, 5×10^3 cells from each culture were seeded on a 96-well plate and allowed to adhere overnight. Cisplatin was added at the indicated concentration. Cell proliferation was monitored in real time using the IncuCyte Live Cell Imaging System (Essen Bioscience), and data was collected every 2 hours. Data analysis was performed using the IncuCyte software using a red and green fluorescence mask to accurately count each cell type. Statistical significance was measured using ANOVA test (two or one-way) and t-test. The media was not refreshed for the duration of the observations.

Conditioned medium assay. Complete growth medium was added to 4×10^6 cells on a 10 cm dish to condition medium. After 24 hours, conditioned medium was collected in a centrifuge tube and spun down at 2500 RPM for 10 mins. Afterwards, conditioned medium was added to 5×10^3 cells seeded on a 96-well plate. Cell proliferation was monitored in real time using the IncuCyte Live Cell Imaging System (Essen Bioscience), and data was collected every 2 hours. Data analysis was performed using the IncuCyte software using a red and green fluorescence mask to accurately count each cell type.

SAHA treatment and drug sensitivity assay. Cells were (3×10^5) plated on a 6 cm dish and allowed to adhere overnight. Fresh medium containing SAHA (0.25 μM/0.5 μM) was added every 24 hours for 3 days. After 3 days, 5×10^3 cells were seeded on a 96-well plate and allowed to adhere overnight. Cisplatin (5 μM) was added, and cell proliferation was monitored in real time using the IncuCyte Live Cell Imaging System (Essen Bioscience). Data was collected every 2 hours, and analysis was performed using the IncuCyte software using a red and green fluorescence mask to accurately count each cell type.

In vivo therapy. NSCLC cell lines H2009 (cisplatin-resistant) and H23 (cisplatin-sensitive) were seeded in a 6-well plate until 60-70% confluency. One day prior to microinjection H2009 and H23 cells were stained with DiI (fluorescent lipophilic cationic indocarbocyanine) green and DiI red dye, respectively. On the day of microinjection, the 48-hpf (hours post fertilization) zebrafish larvae were dechorionated to release the larvae. The larvae were anesthetized using tricaine (MS-222) at a final concentration of 200 μg/ml (stock 5 mg/ml). The larvae were left in anesthetic for 1-2 h until they were motionless for efficient microinjection. The two cell lines were trypsinized and cell number counted using a cell counter (Nexcelom Bioscience Cellometer Auto T4). The two cells were made into a homogenous suspension with 10 cells per nanoliter (nl). Next, the two cell lines were mixed in a 4:1 ratio (H23:H2009). The mix of cells were injected in the perivitelline space (PVS) of anesthetized 48-hpf (hours post fertilization) larvae (184 nl= approx. 184 cells). The 24-hpi (hours post injection) zebrafish xenografts were screened for formation of an obvious bolus of cancer cells (tumor) using a fluorescence microscope. The larvae

were distributed in a 96-well plate with different treatment sets (untreated and drug treated). Drug toxicity effects on growth and development were also assessed by examining the length and shape of the zebrafish body. For the untreated sample set, the larvae were left in embryo media throughout the experiment. For intermittent and continuous samples, 20 μM cisplatin was added at Day 1. For intermittent samples, the drug was removed after three days and then these larvae were continued in only embryo media, while for continuous sample set at three days we replenished them with second dose of 20 μM cisplatin. The larvae were imaged using Zeiss Observer 7 microscope for Day 1, Day 3 and Day 5 of microinjection. The images were processed using FIJI imaging software. Each image was split into different color channels (gray, red and green). Threshold of fluorescent images were determined with Otsu method. The images were converted to binary images. The mean gray value and area were calculated. These values were used to calculate green intensity versus red intensity. The data points were plotted with Prism software. The biological replicates used were 10 samples for intermittent, 8 continuous and 4 untreated samples. The p-values were calculated using one-way ANOVA.

Reference

1. Li, X.-Y.; Pietschke, C.; Fraune, S.; Altrock, P.M.; Bosch, T.C.G.; Traulsen, A. Which games are growing bacterial populations playing? *J. R. Soc. Interface* **2015**, *12*, 20150121, doi:10.1098/rsif.2015.0121.
2. Byrd, R.H.; Lu, P.; Nocedal, J.; Zhu, C. A Limited Memory Algorithm for Bound Constrained Optimization. *SIAM J. Sci. Comput.* **1995**, *16*, 1190–1208, doi:10.1137/0916069.
3. Mohanty, A., Nam, A., Pozhitkov, A., Yang, L., Srivastava, S., Nathan, A., Wu, X., Mambetsariev, I., Nelson, M., Subbalakshmi, A.R., et al., (2020). A Non-genetic Mechanism Involving the Integrin beta4/Paxillin Axis Contributes to Chemoresistance in Lung Cancer. *iScience* 23, 101496.
4. Fiaschi, T.; Chiarugi, P. Oxidative stress, tumor microenvironment, and metabolic reprogramming: A diabolic liaison. *Int. J. Cell Biol.* **2012**, *2012*, 762825, doi:10.1155/2012/762825.
5. Northcott, J.M.; Dean, I.S.; Mouw, J.K.; Weaver, V.M. Feeling Stress: The Mechanics of Cancer Progression and Aggression. *Front. Cell Dev. Biol.* **2018**, *6*, 17, doi:10.3389/fcell.2018.00017.
6. Carvalho, G.; Guilhen, C.; Balestrino, D.; Forestier, C.; Mathias, J.-D. Relating switching rates between normal and persister cells to substrate and antibiotic concentrations: a mathematical modelling approach supported by experiments. *Microb. Biotechnol.* **2017**, *10*, 1616–1627, doi:10.1111/1751-7915.12739.
7. Banelli, B.; Carra, E.; Barbieri, F.; Würth, R.; Parodi, F.; Pattarozzi, A.; Carosio, R.; Forlani, A.; Allemanni, G.; Marubbi, D.; et al. The histone demethylase KDM5A is a key factor for the resistance to temozolomide in glioblastoma. *Cell Cycle* **2015**, *14*, 3418–3429, doi:10.1080/15384101.2015.1090063.
8. Pazarentzos, E.; Bivona, T.G. Adaptive stress signaling in targeted cancer therapy resistance. *Oncogene* **2015**, *34*, 5599–5606, doi:10.1038/onc.2015.26.
9. Ozawa, S.; Sugiyama, Y.; Mitsuhashi, J.; Inaba, M. Kinetic analysis of cell killing effect induced by cytosine arabinoside and cisplatin in relation to cell cycle phase specificity in human colon cancer and Chinese hamster cells. *Cancer Res.* **1989**, *49*, 3823–8.
10. Samuel, P.; Mulcahy, L.; Furlong, F.; McCarthy, H.; Brooks, S.A.; Fabbri, M.; Pink, R.C.; Carter, D.R.F. Cisplatin induces the release of extracellular vesicles from ovarian cancer cells that can induce invasiveness and drug resistance in bystander cells. *Philos. Trans. R. Soc. B: Biol. Sci.* **2018**, *373*, 20170065, doi:10.1098/rstb.2017.0065.
11. Petzold, L. Automatic Selection of Methods for Solving Stiff and Nonstiff Systems of Ordinary Differential Equations. *SIAM J. Sci. Stat. Comput.* **1983**, *4*, 136–148, doi:10.1137/0904010.
12. Soetaert, K., Petzoldt, T., Setzer, R.W., (2010). Solving Differential Equations in R: Package deSolve. **2010**, *33*, 25.
13. Goldberg, D.E. *Genetic Algorithms in Search Optimization and Machine Learning*; Addison-Wesley: Boston, MA, USA, 1989; 432p.

14. Scrucca, L. GA: A Package for Genetic Algorithms inR. *J. Stat. Softw.* **2013**, *53*, 1–37, doi:10.18637/jss.v053.i04.
15. Tönsing, C.; Timmer, J.; Kreutz, C. Profile likelihood-based analyses of infectious disease models. *Stat. Methods Med Res.* **2018**, *27*, 1979–1998, doi:10.1177/0962280217746444.
16. Kreutz, C.; Raue, A.; Kaschek, D.; Timmer, J. Profile likelihood in systems biology. *FEBS J.* **2013**, *280*, 2564–2571, doi:10.1111/febs.12276.
